# Etiological spectrum and contributing factors of male infertility among Pakistani men

**DOI:** 10.12669/pjms.41.12.13163

**Published:** 2025-12

**Authors:** Sarwat Jehan, Amanullah Khan, Musarrat Riaz

**Affiliations:** 1 Sarwat Jehan, *MBBS, MCPS, FCPS (Gynae & Obs)*, Associate professor/Consultant In-charge IVF, Baqai institute of Reproduction and Developmental Sciences, Baqai Medical University, Karachi, Pakistan; 2 Amanullah Khan, *MBBS, FCPS (Med)*, Consultant Physician, Baqai Institute of Diabetology & Endocrinology, Karachi, Pakistan; 3 Musarrat Riaz, *FCPS(Med), FCPS(Endocrine)*, Associate Professor, National Institute of Diabetes & Endocrinology, Dow University of Health Sciences, Karachi, Pakistan

**Keywords:** Azoospermia, Male infertility, Reproductive health, Semen analysis, Varicocele

## Abstract

**Background & Objective:**

Infertility is a significant concern worldwide, affecting millions of couples, yet often under-recognized. In Pakistan, approximately 22% of couples experience infertility, with male factors contributing in 20-25% of cases. Despite advances in reproductive medicine, male infertility remains poorly understood, often requiring specialized interventions. This study aimed to investigate the underlying causes and contributing factors of male infertility in Pakistani men presenting at a specialized infertility unit in Karachi, Pakistan.

**Methodology:**

A retrospective observational study was conducted at the Baqai Institute of Development Sciences (BIRDS), Baqai Medical University, Karachi, from 2015 to 2022. The study included all male patients of reproductive age (18-50 years) who were married, cohabiting and attempting to conceive for over a year without success. For the present analysis, data from couples in whom male factors were identified as the predominant cause of infertility were included. Clinical characteristics, demographic variables, lifestyle habits, comorbid conditions and relevant laboratory parameters were retrieved from medical records. Data were entered and analyzed using SPSS Statistics, version 25.0.

**Results:**

A total of 809 infertile males aged 18-50 years were evaluated. Primary infertility was more common (79.4%) compared to secondary infertility (20.6%). Among the study population, notable comorbidities included diabetes (41.1%) and hypertension (21.3%). Lifestyle factors were also prevalent, with 34.2% reporting smoking, 39.7% using tobacco and 34.7% having a history of testicular surgery. Semen analysis revealed azoospermia in 16.7% of cases, asthenoteratozoospermia in 8.3% and normozoospermia in only 0.4%. A considerable number of participants had a history of sexually transmitted diseases (STDs) and varicocele was identified in 19.4% of cases. Additionally, a family history of infertility was present in 21.9% of individuals.

**Conclusion:**

Male infertility in Pakistan is influenced by a complex interplay of metabolic, infectious and lifestyle factors. Diabetes, sexually transmitted infections and prior testicular surgeries were key contributors. These findings call for greater awareness and accessible fertility services, along with multicenter research to guide health policies and optimize management in resource-limited settings.

## INTRODUCTION

Infertility is a global health issue affecting approximately 15% of couples worldwide, with substantial variation in prevalence across regions.^1^ In Pakistan, approximately 21.9% of the population experiences infertility, with 3.5% attributed to primary infertility and 18.5% to secondary infertility.^2^ Half of these couples will have a component of male factor infertility and almost 30% of infertilities will be caused solely by male factors.^3^ Idiopathic causes of the male infertility accounts for about 50% of all the causes.^4^

Male factor infertility is a general term that describes a situation in which the inability to conceive is associated with an alteration identified in the male partner. This dysfunction may be associated with low sperm concentration (oligozoospermia), poor sperm motility (asthenozoospermia) or abnormal sperm morphology (teratozoospermia); however, generally, a disturbance of all these variables, is mostly frequent in male subfertility.^5^

Unlike female infertility, male infertility is generally underreported, particularly in regions where cultural and social norms in societies hinder the collection of accurate statistics.^5^ In countries like Pakistan, infertility is often attributed to the female partner and there is limited knowledge about the male infertility etiologies.^6^ As a result, men are less likely to consent to fertility evaluations, leading to significant underreporting of male infertility cases.

Over the past few decades, research has highlighted a declining trend in sperm counts, suggesting a worsening male fertility crisis. While early treatment approaches yield limited success, advanced reproductive technologies such as intracytoplasmic sperm injection (ICSI) and in vitro fertilization (IVF) are often necessary.

Despite the prevalence of infertility in Pakistani men, limited data exist regarding its etiology. Without accurate data, it is not possible to identify and comprehensively treat infertile men. This study seeks to examine the causes and risk factors contributing to male infertility in Pakistan, with the goal of improving diagnostic and treatment strategies.

## METHODOLOGY

This retrospective, observational study was conducted at the Baqai Institute of Reproduction and Developmental Sciences (BIRDS), Baqai Medical University over a seven-year period from January 2015 to December 2022. The study included all male patients of reproductive age (18-50 years) who were married, cohabiting and attempting to conceive for over a year without success. For the present analysis, data from couples in whom male factors were identified as the predominant cause of infertility were included. Infertility was classified as either:

### Inclusion Criteria:

- Primary infertility: Inability to achieve conception despite regular unprotected intercourse with no prior history of pregnancy.
- Secondary infertility: Inability to conceive after a prior successful pregnancy.

### Exclusion Criteria:

- Patients with incomplete medical records.
- Patients with known genetic infertility syndromes (e.g., Klinefelter syndrome).
- Individuals who had undergone previous vasectomy or sterilization procedures.
- Cases where female partner infertility was the primary cause, as documented by medical records.

### Ethical considerations:

The study was approved by the institutional ethical review board at Baqai Medical University. (BMU-EC/05-2021, dated November 17, 2021). As this was a retrospective study, informed consent was waived. However, all data were anonymized to ensure patient confidentiality.

Patient medical records were systematically reviewed to collect demographic, clinical and laboratory data. The following variables were analyzed:

Demographic information’s included were age at the time of presentation, duration of infertility, occupation and socioeconomic status. Medical and Surgical History included history of childhood illnesses affecting fertility (e.g., mumps orchitis), Presence of comorbidities such as diabetes, hypertension, obesity, thyroid disorders, hyperprolactenmia and other endocrine abnormalities and Prior surgeries, particularly those affecting the reproductive or urinary system (e.g., varicocele repair, hernia surgery, testicular torsion surgery). History of sexually transmitted infections (STIs) and other urogenital infections were also reviewed. Additionally, family history of infertility or genetic disorders affecting reproductive health as well as lifestyle and environmental Factors like Smoking, alcohol consumption and recreational drug use, occupational exposure to heat, radiation, or industrial toxins and history of excessive physical exertion or sedentary lifestyle. Semen analysis was performed according to World Health Organization (WHO) 2010 guidelines.^7^

Patients were categorized based on semen analysis findings as follows:

- *Oligozoospermia:* Low sperm concentration (<15 million/mL).
- *Asthenozoospermia:* Reduced sperm motility (<40% progressive motility).
- *Teratozoospermia:* Abnormal sperm morphology (<4% normal forms).
- *Azoospermia:* Absence of sperm in semen.
- *Normozoospermia:* Normal semen parameters.

In cases of abnormal semen analysis, serum hormonal assays were conducted, including:

- Follicle-stimulating hormone (FSH), Luteinizing hormone (LH), Testosterone, Prolactin and thyroid function tests (TSH, FT4) (if clinically indicated)

Patients with clinical suspicion of varicocele or testicular abnormalities underwent scrotal ultrasonography with Doppler imaging to assess Testicular size and consistency, Presence and grading of varicoceles, Epididymal and vas deferens abnormalities. Karyotyping for suspected chromosomal abnormalities (e.g., Klinefelter syndrome) were done if indicated.

Microbiological Tests included Urinalysis and urine culture for urinary tract infections, Semen culture in cases with leukocytospermia or suspected infections and Screening for sexually transmitted infections (e.g., Chlamydia, Gonorrhea).

### Statistical analysis:

Data were analyzed using SPSS version 25.0. Descriptive statistics were presented as means (± standard deviation) for continuous variables and percentages for categorical variables. Comparisons between groups (primary vs. secondary infertility, normozoospermic vs. abnormal semen analysis) were conducted using Chi-square tests for categorical variables, Independent t-tests for continuous variables, Logistic regression analysis to assess predictors of male infertility. A p-value <0.05 was considered statistically significant.

## RESULTS

The study included a total of 809 infertile men with the mean age of 32.45±7.61 years. Of these, 642 (79.4%) presented with primary infertility, while 167 (20.6%) had secondary infertility. The majority of patients, 737 exhibited normal secondary sexual characteristics, suggesting that most cases were not due to hormonal deficiencies. Out of 809 patients, 58 (41.13%) were diabetic, 30 (21.28%) were hypertensive, while pulmonary TB cases were reported in 5(3.55%) patients.

Additionally, 25(34.72%) of the participants had undergone testicular surgery, highlighting the potential role of previous surgical interventions in male infertility. A positive family history of infertility was recorded in 122 (21.94%) of cases, while family history of diabetes and hypertension were noted in 121(21.76%) and 58 (10.43%) cases, respectively. History of smoking and tobacco use was documented in 137(34.42%) and 158(39.7%) patients respectively. (Table-I).

**Table-I:** Baseline characteristics of study participants.

Parameters	N=809
**Age, years**	32.45 ± 7.61
**Infertility (n=809)**	
Primary	642 (79.4%)
Secondary	167 (20.6%)
**Co Morbidities**	
Diabetes	58 (41.1%)
Hypertension	30 (21.3%)
Pulmonary TB	5 (3.5%)
**Past history of testicular surgery**	25 (34.7%)
**Family History of Chronic Disorder**	
Diabetes	121 (21.7%)
Hypertension	58 (10.4%)
Infertility	122 (21.9%)
**Type of addiction**	
Smoking	137 (34.2%)
Tobacco	158 (39.7%)

Sexual problems like erectile dysfunction were documented in 19(12.67%), 21(14%) men had premature ejaculation while 3(2%) of men had decreased libido. Urinary tract infections were reported in 33 participants while sexually transmitted diseases (STDs) were reported in 90 infertile men. In Table-II the key findings of semen abnormalities are presented. Semen analysis shows the average volume of 3.35±3.19. Azoospermia was seen in 135(16.7%) cases, Asthenoteratozospermia in 67(8.3%), teratozoospermia in 46 (5.6) while Normozoospermia was observed in only 3(0.4%) patients.

**Table-II:** Semen characteristics of study population.

Parameters	N=809
**Type of Sexual Problem**	n=148
Erectile dysfunction	19 (12.7%)
Premature ejaculation	21 (14%)
Decreased libido	3 (2%)
**Sexually Transmitted Disease (STD)**	90 (11.12%)
**Urinary tract infection**	33 (0.04%)
**Semen volume**	3.35 ± 3.19
**Azoospermia**	135 (16.7%)
**Asthenoteratozospermia**	67 (8.3%)
**Teratozoospermia:**	46 (5.6)
**Normozoospermia**	3 (0.4%)

## DISCUSSION

Our study highlights the considerable burden of primary infertility among Pakistani men. In our cohort, 79% of infertile men were affected by primary infertility, whereas 20% experienced secondary infertility. These figures closely align with the results of a meta- analysis focusing on studies involving infertile Iranian couples.^8^ Additionally, our findings are consistent with a local study conducted in Pakistan by Irfan M. et al.^2^

Diabetes emerged as a major comorbidity, aligning with previous research that links metabolic disorders to impaired spermatogenesis and hormonal imbalances. Results reveals that 58 (41.13%) of infertile men were diagnosed with diabetes. Therefore, uncontrolled diabetes may be considered a potential contributing factor to male infertility.^9^ The high prevalence of hypertension further underscores the role of cardiovascular health in male fertility. Our study correlates with the findings of one the western studies in which hypertension was reported to be common among the infertile males.^10^

Comparing our findings with national, regional and international data, Pakistani men exhibit a higher prevalence of diabetes-associated infertility than reported in some neighboring countries such as India and Bangladesh.^11^ This discrepancy may be attributed to genetic predisposition, dietary habits and access to healthcare services. In contrast, Western studies have reported a lower impact of diabetes on male infertility, possibly due to better metabolic control and lifestyle modifications.^12^ Our findings indicates that males with an elevated risk of experiencing infertility have an average age of 32.4 years. This observation aligns with global analysis^13^ that specifically emphasize a heightened prevalence of infertility among individuals aged 30-34.

Testicular surgery history was noted in 34.72% of our patients, which is notably higher than the 15-20% reported in Middle Eastern and South Asian populations.^4^,^5^ This difference could be attributed to delayed diagnosis and limited awareness regarding male reproductive health in Pakistan. Family history of infertility, observed in 21.94% of cases, also reflects genetic influences, aligning with global studies that highlight hereditary factors contributing to infertility.^5^

One of the interesting finding observed during our investigation was 90 (11,12%) of infertile men were positive for STDs. Nevertheless, another study from Africa found that around 46% of Sub-Sahara African men have infertility related to sexually transmitted diseases so there is a strong correlation among the infertility and STDs.^13^ According to existing literature, varicocele is considered a major etiological factor of male infertility leading to compromised sperm quality, sperm production and viability as abnormal dilation of veins in scrotum results in increased temperature. Our result shows that 52(19.4%) of infertile men were positive for varicocele whereas 216(80.6%) cases were diagnosed negative, showing a contrasting effect within the observed population.^14^

Globally the sperm count and quality decline have been widely reported in developed nations, particularly in Europe and North America.^15^ The decline is often linked to environmental factors, lifestyle changes and increased exposure to endocrine-disrupting chemicals. While similar trends are being observed in Pakistan, there is limited data on impact of environmental toxins on male fertility.

Despite the extensive data available on male infertility globally, conflicting reports exist regarding its prevalence and causes in Pakistan. This study highlights the necessity of conducting more extensive, multi-centric research to generalize findings and enhance fertility management strategies.

### Limitations:

However, there are some limitations of our study. The study was conducted at a single tertiary care center, which may restrict the generalizability of the findings to the wider Pakistani population. Additionally, the retrospective design limited the ability to establish causal relationships between identified risk factors and infertility. Some variables, such as lifestyle habits and past medical history, relied on patient self-report, introducing the possibility of recall bias. Female factors contributing to infertility were not explored in detail, as the focus was restricted to male infertility. As a hospital-based study, the findings may not fully represent community-level patterns of male infertility.

## CONCLUSION

Male infertility in Pakistan is influenced by a complex interplay of metabolic, infectious and lifestyle factors. Diabetes, sexually transmitted infections and prior testicular surgeries were key contributors. These findings call for greater awareness and accessible fertility services, along with multicenter research to guide health policies and optimize management in resource-limited settings.

### Future recommendations:

1. ***Early screening:*** Infertility workups should be standardized for early detection of male infertility risk factors.
2. ***Lifestyle modifications:*** Public health initiatives should emphasize lifestyle changes to reduce diabetes and hypertension-related infertility.
3. ***Genetic research:*** Further studies are required to explore genetic links to male infertility in the Pakistani population.
4. ***Advanced reproductive technologies*:** Increased accessibility to ARTs such as ICSI and IVF should be prioritized for severe male infertility cases.

### Authors’ Contribution:

**SJ:** Principal investigator, designing the study, Data collection and compilation, Manuscript writing, statistical analysis, Literature search, Final approval, responsible for accuracy and integrity of work.

**AK:** Literature search, Data collection and compilation, Manuscript writing, statistical analysis.

**MR:** Proposed the idea, designed the study, interpretation of data, reviewed the manuscript, statistical analysis, literature search, supervision, final approval.
